# Association of gender and main comorbidities with expression of lncRNAs and mRNAs in COVID-19 patients

**DOI:** 10.1016/j.jgeb.2025.100650

**Published:** 2025-12-24

**Authors:** Hassan Abolghasemi, Hamidreza Kheiri, Hamid Sedighian, Elham Behzadi, Reza Kachuei, Mozhgan Kheirandish, Masoud Arabfard, Abbas Ali Imani Fooladi

**Affiliations:** a Applied Microbiology Research Center, Biomedicine Technologies Institute, Baqiyatallah University of Medical Sciences, Tehran, Iran; b Academy of Medical Sciences of the I.R. of Iran, Tehran, Iran; c Molecular Biology Research Center, Biomedicine Technologies Institute, Baqiyatallah University of Medical Sciences, Tehran, Iran; d Artificial Intelligence in Health Research Center, Biomedicine Technologies Institute, Baqiyatallah University of Medical Sciences, Tehran. Iran

**Keywords:** Gene expression, lncRNA, COVID-19, Protein-Protein Interaction Networks, Bioinformatics

## Abstract

**Background:**

SARS-CoV-2 causes mortality in a considerable number of patients with COVID-19. The association of comorbidities and gender with the expression of lncRNAs and mRNAs in COVID-19 patients is not fully understood. The purpose of the present study was to explore this association.

**Method:**

We used Transcriptomics data for lncRNAs and mRNAs from the integrated Gene Expression Omnibus (GEO) to identify Differentially Expressed Genes (DEGs) using R software for statistical and data analysis. Then, we carried out Gene Ontology (GO) analysis and constructed a Protein-Protein Interaction (PPI) network to identify interactions between the genes.

**Results:**

In this study, we divided samples into four groups and compared Differentially Expressed lncRNAs (DEls) and DEGs. Genes enriched in immune response and cytokine pathways were identified by GO analysis. By considering the protein–protein interaction network, the hub genes were ALAS2, CCL2, AHSP, and IL5.

**Conclusion:**

mRNAs and lncRNAs could be used to identify the effects of SARS-CoV-2 on defined parameters (such as gender, main comorbidities in recovery, and treatment stages). Heme/hemoglobin metabolism was enriched in groups 1, 2, and 4, with four common genes (*ALAS2*, *AHSP*, *HBD*, and *CA1*) that are associated with the immune response to infection. *CCL2* was enriched in group 3 and its expression was remarkably high in patients with an unfavorable outcome compared to other cases. Also, while both *IL-5* and *ALAS2* were enriched in group 4, IL-5 appeared to have no significant role in COVID-19. Overall, we conducted a bioinformatics analysis to predict how mRNAs and lncRNAs interact in patients with different characteristics such as gender, underlying disease, and treatment or recovery stages. mRNAs and lncRNAs can be potential biomarkers to examine the effect of SARS-CoV-2 on defined parameters.

## 1 Introduction

The large Coronaviridae family infects both animals and humans. Coronavirus disease 2019 (COVID-19) was first reported in Wuhan, China, in December 2019 and then threatened public health worldwide.^1^, ^2^, ^3^ Its causative agent, severe acute respiratory syndrome coronavirus 2 (SARS-CoV-2), is a single-stranded RNA virus that belongs to the same Coronaviridae family as the viruses that caused Severe Acute Respiratory Syndrome (SARS) in 2003 and Middle East Respiratory Syndrome (MERS) in 2012.^4^, ^5^ As of March 13, 2023, the World Health Organization (WHO) documented a total of 6,811,920 deaths attributed to COVID-19, along with numerous reported cases.^6^

When SARS‐CoV‐2 enters respiratory epithelial cells, the immune system responds by producing inflammatory cytokines and suppressing interferon (IFN). Due to an increased level of pro-inflammatory cytokines in the blood, macrophages, and neutrophils infiltrate into lung tissue and a cytokine storm occurs. Cytokine storm is strongly associated with severe stages of COVID-19 such as lung injury and multi-organ failure.^7^, ^8^, ^9^, ^10^ Development and activation of immune cells rely on dynamic regulation of gene expression through transcriptional and post-transcriptional mechanisms^11^, in which non-coding RNAs play an important role. Long non-coding RNAs (lncRNAs) and short non-coding RNAs (sncRNAs) are two classes of non-coding RNAs.^12^, ^13^ According to genome-wide association studies (GWAS), dysregulation of lncRNAs, particularly that involving single nucleotide polymorphisms (SNPs), is responsible for various human diseases.^14^ lncRNAs are key regulators of inflammatory genes and crucial players in regulating inflammatory responses.^15^, ^16^, ^17^

MicroRNAs (miRNAs) are single-stranded noncoding RNAs that suppress the expression of protein-coding genes by inducing translation repression, mRNA degradation, or both.^18^

The integrated analysis of mRNA, lncRNA, and miRNA sequences will enhance our knowledge of the biological functions and signaling pathways associated with Differentially Expressed Genes (DEGs).^19^ Due to the role of lncRNAs as anti-viral inflammatory response regulation in host cells, their co-expression with human genes also contributes to immunologic responses during SARS-CoV-2 lung infection. In this respect, detecting mRNA-based and lncRNA-based biomarkers allows us to effectively discriminate between milder cases and those at higher risks of mortality.^20^

Early epidemiological studies identified age and underlying medical comorbidities as reliable predictors for estimate the severity of COVID-19.^21^, ^22^, ^23^, ^24^ The contribution of gender to SARS-CoV-2 infection offers an essential understanding of both biological mechanisms and illness outcomes. Although men and women show similar rates of infection, the risks of severe illness and death are higher in men^25^, ^26^, ^27^. This discrepancy is evident in data collected by the WHO, which indicates a nearly equal gender distribution among confirmed cases (gender ratio of M: F cases = 1.03:1).^28^ Nonetheless, in many nations, the mortality rate among confirmed cases is higher in men than in women. This study tries to discover how the expression of lncRNA and mRNA correlates with gender and main comorbidities.

## 2 Materials and methods

### 2.1 Data preparation

To acquire the baseline expression levels of both long non-coding RNAs (lncRNAs) and messenger RNAs (mRNAs), transcript per million (TPM) values of gene expression were extracted from the dataset GSE157859, accessible through the Gene Expression Omnibus (GEO). The raw sequencing data libraries for both lncRNA and mRNA were acquired from a total of 38 samples under the GEO accession ID “GSE157859″. All data were downloaded from the SRA database under the accession ID ”SRP282150″. This dataset contains samples of peripheral blood mononuclear cells (PBMC) of patients with COVID-19 symptoms during the treatment and recovery stages (convalescence and rehabilitation). Table 1 provides the details regarding gender and main comorbidities to facilitate a comparison between the treatment and recovery groups. Main comorbidities are defined as the presence of one or more additional health conditions that coexist alongside the primary disease of interest, which in this case is COVID-19. The samples were derived from patients with various main comorbidities, including hypertension, hepatitis C virus infection, type II diabetes, and myocardial infarction.^29^

**Table 1:** A Sample key file comparing gender and main comorbidities in the treatment and recovery groups.

Sample	Gender	Main comorbidities	Group
lnc_46	male	no	Treatment
lnc_19	male	no	Treatment
lnc_25	male	no	Treatment
lnc_35	male	Type II diabetes, HCV	Treatment
lnc_44	male	no	Treatment
lnc_24	male	Type II diabetes	Treatment
lnc_36	male	Hypertension	Treatment
lnc_30	male	Hypertension	Treatment
lnc_11	female	no	Treatment
lnc_40	female	no	Treatment
lnc_6	female	Hypertension	Treatment
lnc_8	female	no	Treatment
lnc_16	female	no	Treatment
lnc_31	female	no	Treatment
lnc_14	female	Hypertension	Treatment
lnc_47	male	no	Recovery
lnc_21	male	no	Recovery
lnc_20	male	no	Recovery
lnc_26	male	no	Recovery
lnc_27	male	no	Recovery
lnc_34	male	Type II diabetes, HCV	Recovery
lnc_45	male	no	Recovery
lnc_23	male	Type II diabetes	Recovery
lnc_37	male	Hypertension	Recovery
lnc_38	male	hypertension, myocardial infarction operation	Recovery
lnc_39	male	hypertension, myocardial infarction operation	Recovery
lnc_29	male	Hypertension	Recovery
lnc_28	male	Hypertension	Recovery
lnc_10	female	no	Recovery
lnc_41	female	no	Recovery
lnc_5	female	Hypertension	Recovery
lnc_7	female	no	Recovery
lnc_9	female	no	Recovery
lnc_17	female	no	Recovery
lnc_18	female	no	Recovery
lnc_33	female	no	Recovery
lnc_13	female	Hypertension	Recovery
lnc_15	female	Hypertension	Recovery

In this study, samples were allocated into four groups, and comparisons were made to identify differentially expressed lncRNAs (DEls) and DEGs between these groups. The sample groups were as follows: 1) Men versus women in the treatment group; 2) Men versus women in the recovery group; 3) Individuals with underlying diseases including diabetes, hepatitis C virus (HCV), and hypertension versus individuals without underlying disease in the treatment group; 4) Individuals with underlying disease versus individuals without underlying disease in the recovery group.

### 2.2 Differential expression analysis

Initially, the assessment of data quality was performed using FastQC v.0.11.9, followed by adapter removal using *Trimmomatic* v.040; subsequently, the quality was assessed again by *FastQC*. Data consisted of two sets of outputs which were single-end (SE) and paired-end (PE) files. In the following, mapping was carried out for SE and PE files using STAR v.2.7.8a against the reference genome, GRCH38, which was downloaded from NCBI Assembly. For reads quantification, transcripts assembly was performed using *StringTie* v2.2.0 with the *GENCODE* v33 reference transcriptome. lncRNAs were identified using available databases of *RefLnc* and *LNCipedia*. In the next step, the *DESeq2* package in R software (v3.14) was used to identify DEGs and DEls across the defined comparison groups (e.g., male vs. female patients at different clinical stages). Finally, for each comparison, the *pheatmap* package in R was employed to draw the heatmap plot for the genes with the lowest *P*-value.

### 2.3 Gene and transcript selection criteria

Differentially expressed lncRNAs and mRNAs were identified through an unbiased genome-wide screening approach, which involved analyzing their expression profiles based on stringent statistical criteria. Specifically, we focused on genes exhibiting a P-value <0.05 and a |Log2 Fold Change| ≥ 1, to ensure the selected candidates with both statistically significant and biologically relevant. Although the screening process itself was hypothesis-free, we subsequently highlighted specific genes that passed the thresholds based on their established or emerging roles in inflammatory signaling, heme metabolism, or immune response in viral infections. These genes were not pre-selected but were interpreted post hoc in the context of existing biological knowledge to better understand their relevance to COVID-19 pathophysiology.

### 2.4 Functional enrichment analysis

The Enrichr database was used to perform the functional enrichment analyses in each group. Using ggplot2 (version 3.3.3), we analyzed Gene Ontology (GO) based on cellular component (CC), molecular function (MF), biological process (BP), COVID-related gene set (RG), and pathway enrichment analysis.

### 2.5 Construction and analysis of protein-protein interaction (PPI) network

The functional PPI network of the DEGs and DEls was obtained and visualized using the *Cytoscape stringApp*, and PPI pairs were extracted with a combined score >0.4.

## 3 Results

Using the GSE157859 dataset, we assessed the expression profiles of DEGs and DEls in COVID-19 patients during the treatment and recovery stages. The characteristics of samples within each group are shown in Table 1. The results of the heatmap plot, functional enrichment analysis, and PPI interaction networks are provided for each group.

### 3.1 Specific observations regarding treatment groups

**Fig. 1:**
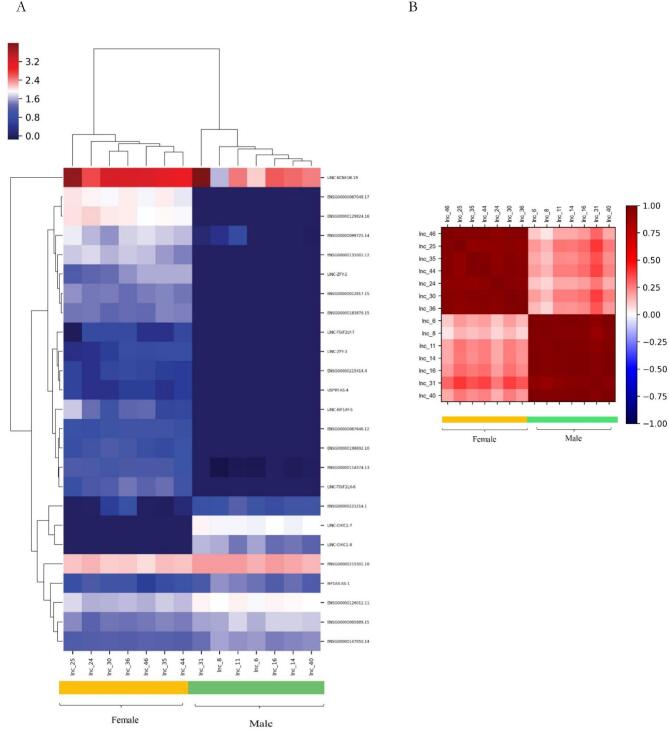
Male vs. female comparison in the treatment group; (A) the heatmap for the differential expression genes with *P*-value <0.05 plotted using the pheatmap package. (B) Correlation between gender and expression of lncRNAs. Columns represent samples and rows represent specific genes. The color scale of the heatmap indicates the expression values.

The results of the heatmap plot of the genes are presented in Fig. 1A. DEG analysis for lncRNA revealed that the expression pattern of lncRNA-mRNA is meaningfully different between males and females (Fig. 1A). To assess the association between the lncRNAs expression and gender in studied COVID-19 cases, the Pearson's correlation test was performed. The results showed no significant correlation between males and females in terms of lncRNAs expression levels in the treatment group (Pearson's correlation coefficient: r = 0.2576, CI = −0.1532 to 0.5924, confidence interval (CI) = 95 %, *P*-value = 0.2139, significance level: correlation coefficient <0.05) (Fig. 1B). These results indicate a relative but non-significant positive correlation between expression patterns of males and females, suggesting that lncRNAs exhibit different expression patterns based on gender during the treatment stage.

**Fig. 2:**
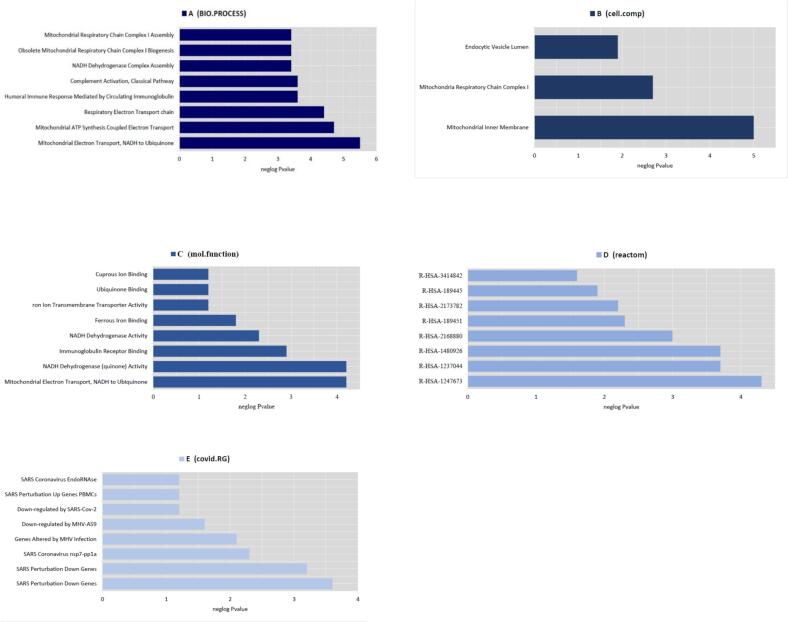
Functional enrichment analyses of DEls and DEGs based on the Enrichr database. The biological processes, cellular components, molecular function, pathways, and COVID-related gene enrichment analyses were depicted in the A, B, C, D, and E, respectively.

**Fig. 3:**
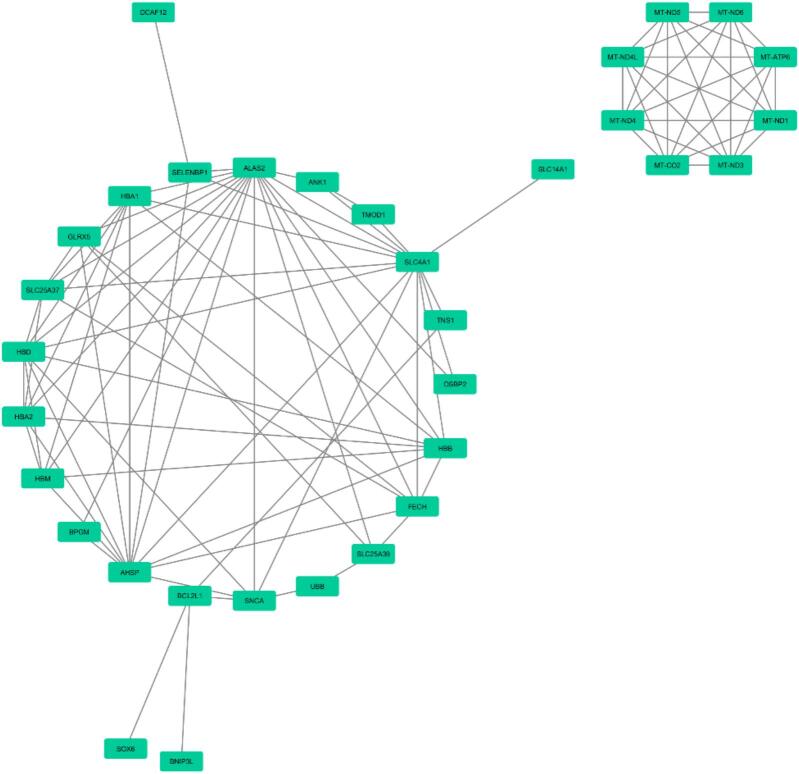
The protein–protein interaction network for DEls and DEGs was constructed using Cytoscape stringApp.

Functional enrichment analysis with the aspects of CC, MF, BP, COVID RG, and Reactome was performed using Enrichr to determine the interactions of DEGs and DEls. According to the results obtained, the top enriched BP term was mitochondrial electron transport from NADH to ubiquinone (*P*-value: 5.71E-06), the most significantly enriched CC term was mitochondrial respiratory chain complex I (*P*-value: 1.05E-05), the top enriched MF term was NADH dehydrogenase (quinone) activity (*P*-value: 6.26E-05), the most enriched pathway was *R-HSA-1247673* (erythrocytes take up oxygen and release carbon dioxide) in *Homo sapiens* (*P*-value: 5.08E-05), the top COVID RG was SARS Perturbation Down Genes Mouse Lung from GSE68820:GPL7202:2 (*P*-value: 2.87E-04). The results of this analysis are presented in Fig. 2A–E. The PPI network with 33 nodes and 95 edges was constructed based on the *Cytoscape stringApp* (Fig. 3).

### 3.2 Specific Observations regarding recovery groups

**Fig. 4:**
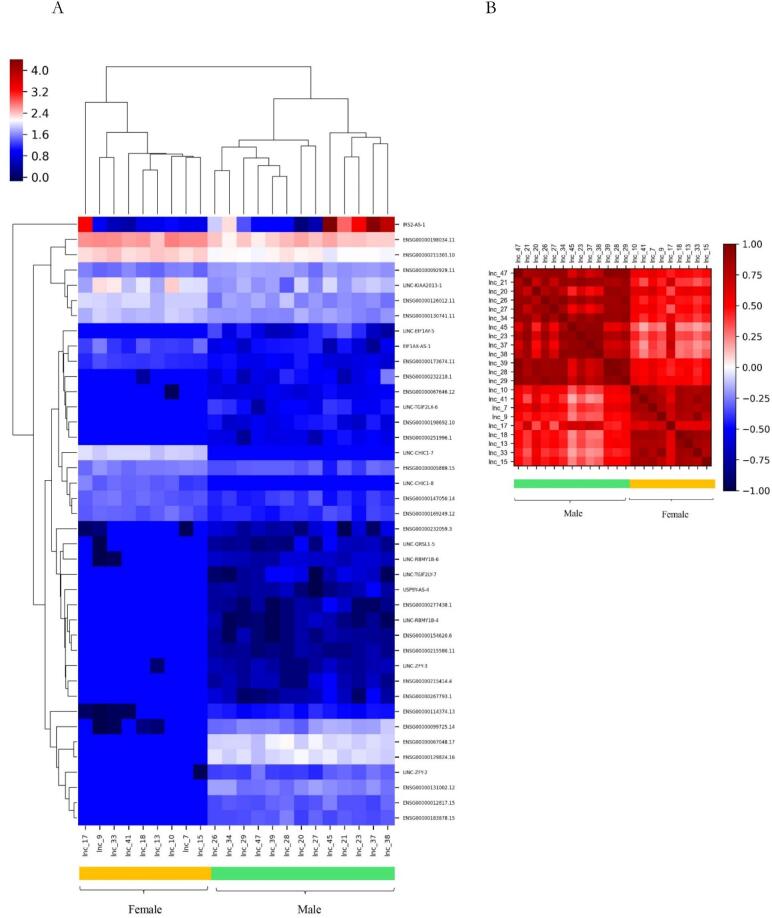
Male vs. Female comparison in the recovery group; (A) the heatmap for the differential expression genes with *P*-value <0.05 plotted using the pheatmap package. (B) Correlation between gender and expression of lncRNAs. Columns represent samples and rows represent specific genes. The color scale of the heatmap indicates the expression values.

The results of the heatmap plot of the genes are presented in Fig. 4A. DEG analysis for lncRNA revealed that the expression pattern of lncRNA-mRNA is different between males and females in the recovery group. The association between the expression of lncRNAs and gender in the recovery group was evaluated using Pearson's correlation test. Results demonstrated that there is a significant correlation between male and female and lncRNAs expression levels in the recovery group (Pearson's correlation coefficient: r = 0.6030, CI = 0.3590 to 0.7699, confidence interval (CI = 95 %, *P*-value = 0.0001, significance level: correlation coefficient <0.05)) (Fig. 4B). These results indicated a significant positive correlation, suggesting that lncRNAs exhibit a coordinated expression patterns between males and females during the recovery stage.

**Fig. 5:**
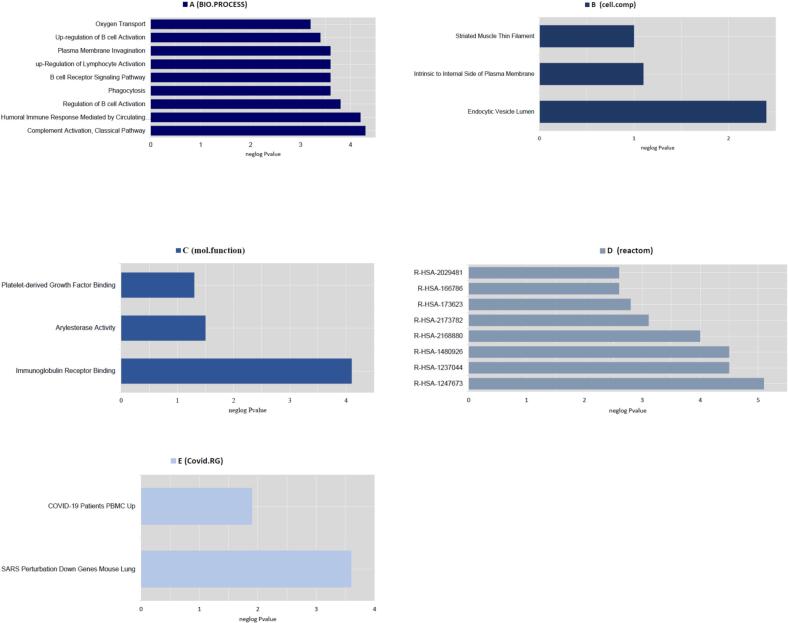
Enrich database was performed to obtain functional enrichment analyses of DEls and DEGs. The top momentous enriched terms are (A) biological processes (B) cellular components (C) molecular function (D) pathways and (E) COVID-related genes.

**Fig. 6:**
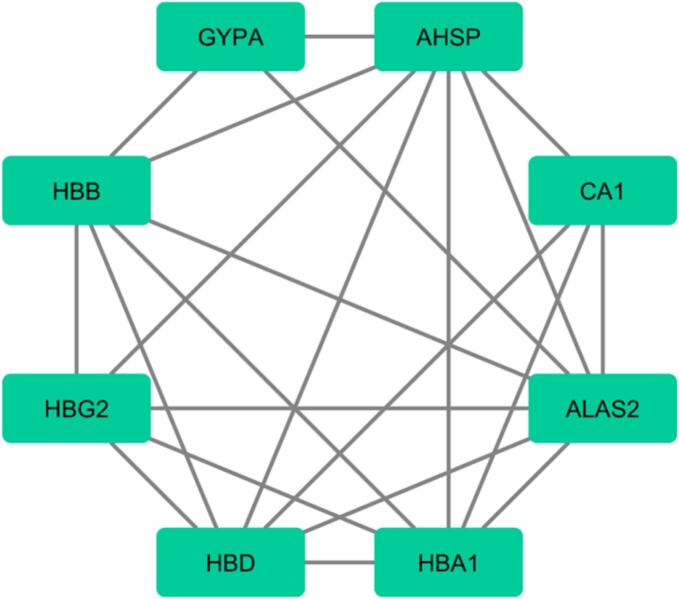
Based on Cytoscape stringApp, the protein–protein interaction network for DEls and DEGs was constructed.

The functional enrichment analysis showed that the top enriched BP term was complement activation in classical pathway (*P*-value: 5.21E-05), the most significantly enriched CC term was endocytic vesicle lumen (*P*-value: 0.004102461), the top enriched MF term was immunoglobulin receptor binding (*P*-value: 7.40E-05), the top enriched pathway was *R-HSA-1247673* (erythrocytes take up oxygen and release carbon dioxide) in *Homo sapiens* (*P*-value: 8.18E-06), the top COVID RG was SARS Perturbation Down Genes Mouse Lung from GSE68820:GPL7202:3 (*P*-value: 0.006284729) (Fig. 5). The PPI network containing 8 nodes and 22 edges was constructed based on the Cytoscape stringApp (Fig. 6).

### 3.3 Comparing the impact of comorbidities on the treatment group

**Fig. 7:**
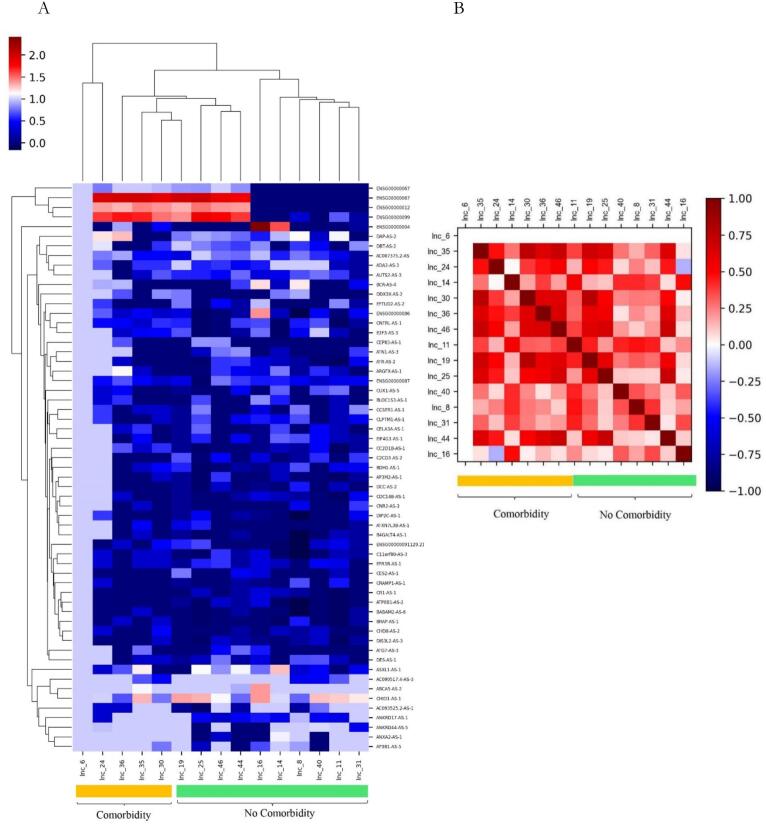
Comparison between individuals with and without underlying disease in the treatment group; (A) the heatmap for differential expression genes with *P*-value <0.05 plotted through the pheatmap package. (B) Correlation between male and female and expression levels of lncRNAs in the recovery group. Columns, rows, and the color scale represent samples, specific genes, and expression values, respectively.

The results of heatmap plot analysis for the genes are demonstrated in Fig. 7A. DEG analysis for lncRNA revealed that the expression pattern of lncRNA-mRNA is different between individuals with and without underlying diseases as it is observable in the heatmap of the treatment group (Fig. 7A). Evaluating the association between the expression of lncRNAs and underlying diseases in the treatment group was done using the Pearson correlation test. Results demonstrated that there is a significant correlation between individuals with and without underlying diseases and expression levels of lncRNAs in the treatment group (Pearson's correlation coefficient: r = 0.8469, CI = 0.7544 to 0.9064, confidence interval (CI = 95 %, *P*-value < 0.0001, significance level: correlation coefficient < 0.05)) (Fig. 7B). These results indicate a strong positive correlation, suggesting that lncRNAs exhibit highly similar expression patterns based on the existence of underlying diseases during the treatment stage.

**Fig. 8:**
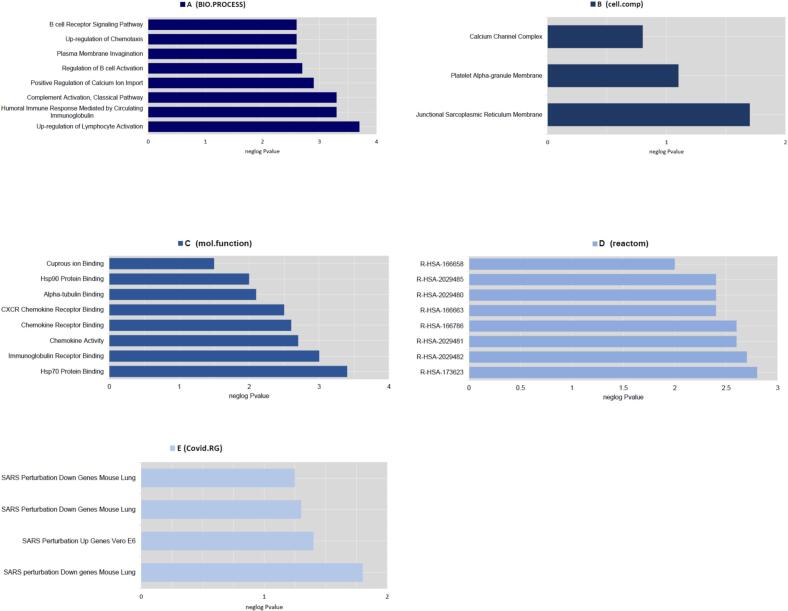
Functional enrichment analyses of DEls and DEGs based on the Enrichr database. The top significant enriched (A) biological processes (B) cellular component (C) molecular function (D) pathways and (E) COVID-related gene set terms are presented.

**Fig. 9:**
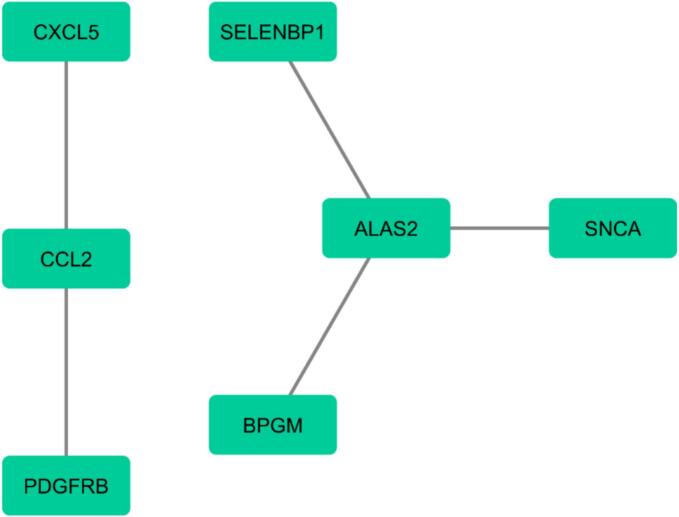
Based on cytoscape stringApp, the protein–protein interaction network for DEls and DEGs was constructed.

Based on functional enrichment analysis, the top enriched BP term was positive regulation of lymphocyte activation (*P*-value: 2.31E-04), the top enriched CC term was junctional sarcoplasmic reticulum membrane (*P*-value: 0.046710082), the top enriched MF term was HSP70 protein binding (*P*-value: 3.86E-04), the top enriched pathway was *R-HSA-173623* (classical antibody-mediated complement activation) in *Homo sapiens* (*P*-value: 0.001530727), the top COVID RG was COVID-19 patients PBMC gene (*P*-value: 0.012819533) (Fig. 8). The PPI network including 7 nodes and 5 edges was constructed based on the Cytoscape stringApp (Fig. 9).

### 3.4 Comparing the impact of comorbidities on the recovery group

**Fig. 10:**
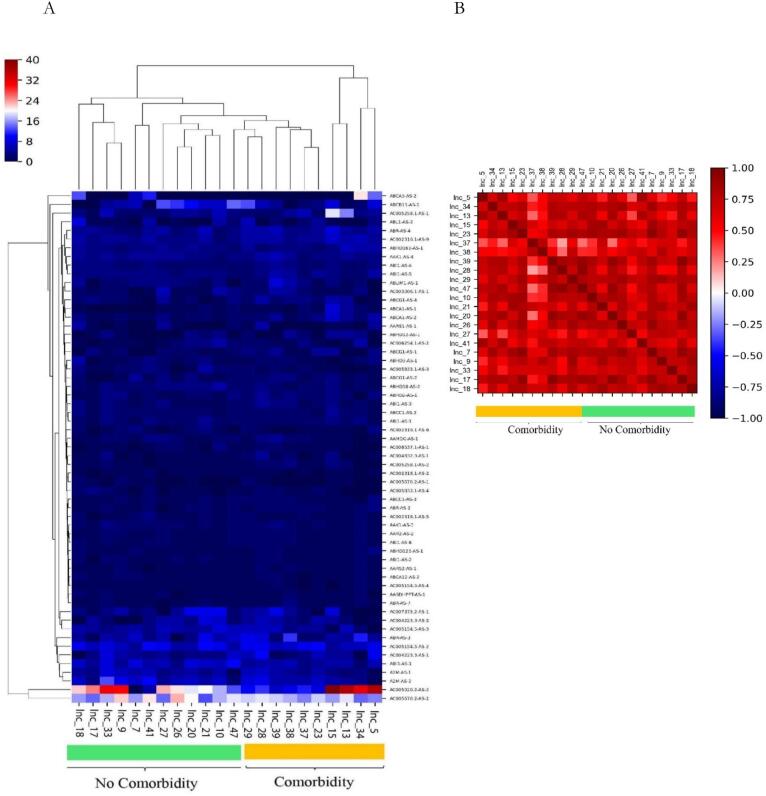
Comparison between individuals with and without underlying disease in the recovery group; (A) the heatmap for the differential expression genes with *P*-value <0.05 plotted using the pheatmap package (B) correlation between male and female and expression levels of lncRNAs in the recovery group. Columns represent samples and rows represent specific genes. The color scale represents the expression values.

The results of the heatmap plot of the genes are shown in Fig. 10 A. DEG analysis for lncRNA revealed that the expression pattern of lncRNA-mRNA is different between individuals with and without underlying diseases as it is observable in the heatmap of recovery (Fig. 10).

The association between the expression of lncRNAs and underlying diseases in the recovery group was assessed using Pearson's correlation test. Results showed that there is no significant correlation between the existence of underlying diseases and expression levels of lncRNAs in the recovery group. The results also demonstrated that there is a significant correlation between individuals with and without underlying diseases and expression levels of lncRNAs in the recovery group (Pearson's correlation coefficient: r = 0.9689, CI = 0.9481to 0.9815, confidence interval (CI = 95 %, *P*-value < 0.0001, significance level: correlation coefficient < 0.05)) (Fig. 10B). These results indicated a positive correlation between the expression patterns of lncRNAs and the existence of comorbidity, so lncRNAs show relatively similar expression patterns based on the existence of underlying diseases in the recovery stage.

**Fig. 11:**
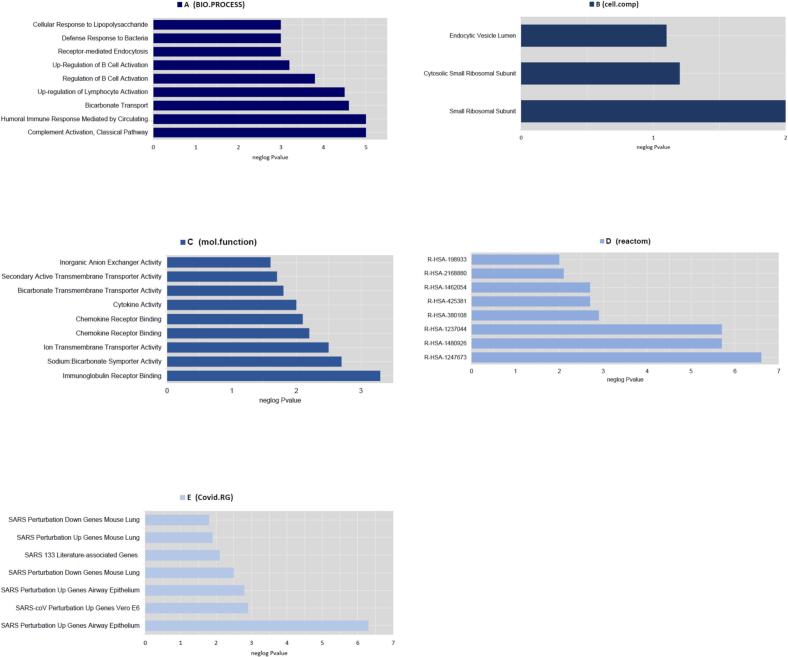
Functional enrichment analyses of DEls and DEGs can be seen based on the Enrichr database. The top significant enriched (A) biological processes (B) cellular components (C) molecular function (D) pathways and (E) COVID-related genes are presented.

**Fig. 12:**
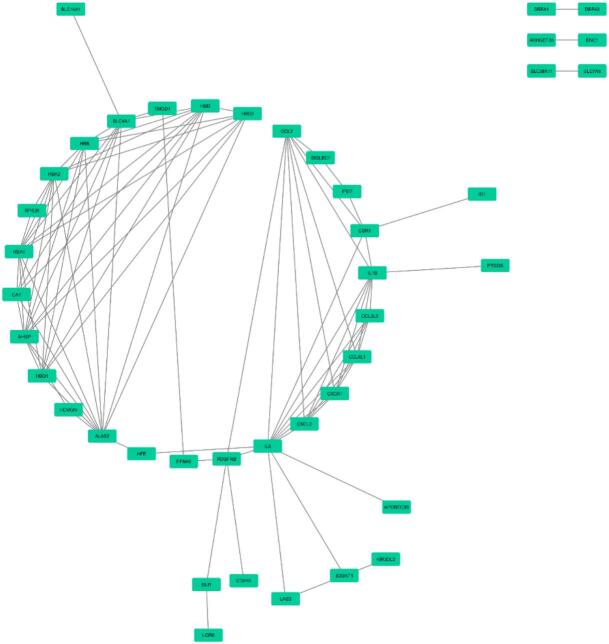
Protein-protein interaction network was constructed for DEls and DEGs using Cytoscape stringApp.

Through functional enrichment analysis, the top enriched BP term was complement activation, classical pathway (*P*-value: 9.94E-06), the top enriched CC term was endocytic vesicle lumen (*P*-value: 0.009936779), the top enriched MF term was immunoglobulin receptor binding (*P*-value: 6.20E-04), the top enriched pathway was R-HSA-1247673 (erythrocytes take up oxygen and release carbon dioxide) in *Homo sapiens* (*P*-value: 3.35E-07), the top COVID RG was SARS-CoV perturbation Up Genes Vero E6 from GSE30589:GPL570:3 (*P*-value: 0.001449643) (Fig. 11). The PPI network contains 42 nodes and 94 edges were constructed based on the Cytoscape stringApp (Fig. 12).

## 4 Discussion

SARS-CoV-2 has infected millions of people around the world and threatened many lives thus far. Hence, timely responses from researchers are essential to confront and control the spread of the COVID-19 pandemic. In fact, updating the safety protocols and intervention guidelines are crucial for controlling all natural infectious diseases. In this study, we examined the association of gender and main comorbidities with the expression of lncRNAs and mRNAs in COVID-19 patients. For this purpose, we analyzed the samples of PBMC in the treatment and recovery stages based on the GSE157859 dataset.

We assessed the expression profile of DEls and DEGs in 4 categories of samples. This categorization allocated the samples to one of the following groups: (1) The treatment group; (2) The recovery group, (3) Patients with and without main comorbidities in the treatment group; and (4) Patients with and without main comorbidities in the recovery group. Thereafter, we performed a functional enrichment analysis of DEGs on the dataset and constructed a PPI Network to detect hub genes.

This assessment revealed that gender and comorbidity cause slight differences in the expression pattern of lncRNAs and mRNAs. Although the correlation test revealed that in the treatment stage of COVID-19, different expression profiles are observable in males in comparison with females, it seems that the overall expression pattern of lncRNAs and mRNAs is similar in males and females in both recovery stages. Similarly, expression profiles of lncRNAs and mRNAs do not show different patterns in the presence of underlying diseases or their absence in both recovery and treatment stages. This supports the theory of different responses to COVID-19 in the early stages of infection. Therefore, a study comparing the expression profiles of lncRNA and mRNA in males and females during treatment and earlier stages of infection is suggested. In addition, the presence or absence of underlying diseases may not be as significant as the specific type of disease, since underlying conditions can range from mild to severe and may cause different expression profiles. Based on this, studying the effect of different types of underlying diseases in the context of COVID-19 infection is also suggested.

The PPI network modules were proposed for group 1 with the high-degree nodes of *ALAS2*, *SLC4A1*, and *AHSP*. *ALAS2* (degree = 16) is the core of module 1. In group 2, female/male COVID-19 patients in the recovery group, the PPI network showed that *ALAS2*, *AHSP*, *HBD*, *HBB*, and *HBA1* were genes with high upregulation. *ALAS2* (degree = 7) and *AHSP* (degree = 7) were the hub genes. In group 3, patients with and without comorbidities, results showed that *ALAS2* and *CCL2* (C–C Motif Chemokine Ligand 2) had high degrees in the PPI network. Besides, in group 4, IL5 (Interleukin 5) and ALAS2 were the nodes with a high degree.

Our enrichment results demonstrated that the genes involved in the heme/hemoglobin metabolism pathway including *ALAS2* (5′-aminolevulinate synthase 2), *AHSP*, *HBA*, and *CA1* were enriched in patients in 1, 2, and 4 groups. *ALAS2* encodes an enzyme responsible for controlling the first step in heme biosynthesis.^30^ ALA-synthase is essential for the development of erythroblasts (red blood cells) and the production of heme during late erythropoiesis, indicating an association with hematological and iron (heme) regulation during infection. *AHSP* stabilizes the normal amounts of hemoglobin by binding to alpha globin.^31^, ^32^ *HBD* encodes the δ-globin and forms the tetramer HbA2 with the α-globin.^33^ Moreover, *CA1* regulates the affinity of hemoglobin for oxygen.^34^ Leit et al. indicated that these genes play a vital role in the cytoprotection of non-erythrocyte cells in response to various stress situations.^35^ Some studies have shown that increased expression of these genes could potentially correlate with the extent of immune cells' response to adverse conditions like inflammation and infection.^36^, ^37^

As previously stated, acute respiratory distress syndrome (ARDS) is a consequence of cytokine storm in SARS-CoV infection. Therefore, the production of large amounts of pro-inflammatory cytokines (*IFNγ, IFNα, IL-1β, IL-6, IL-12, IL-18, IL-33, TNFα*, and *TGFβ*) and chemokines (*CCL2, CCL3, CCL5, CXCL10, CXCL8,* and *CXCL9*) contributes to the inflammatory response.^1^, ^7^ Williams et al. demonstrated several chemokines are involved in the chemokine network in the inflamed lung, including *CXCL10, CCL2*, and *CCL7*.^38^ A combination of immune responses such as interleukin, interferon, colony-stimulating factors, chemokines, and TNF-alpha synthesis is responsible for the development of cytokine storm.^39^ In this regard, dendritic cells and macrophages produce higher levels of cytokines and chemokines in SARS-CoV cases.^40^ Conversely, IFNβ is inhibited in SARS-CoV infections in response to a moderate up-regulation of pro-inflammatory cytokines (like *IL-6* and *TNF-α*) and a dramatic up-regulation of inflammatory chemokines (including *CCL2, CCL3, CXCL10*, and *CCL5*).^41^ In this study, *CCL2, CXCL2,* and *CXCL5* were common chemokines observed in the PPI network groups 3 and 4. Chemokines belong to the cytokine family. *CCL2* and *CXCL2* are inflammatory chemokines that are produced in high concentrations during infection or injury and regulate the migration of inflammatory cells into injured tissues.^42^ The *CCL2* is an essential chemokine in the migration and infiltration of monocytes/macrophages. Cameron et al. indicated that the peripheral blood of SARS patients showed elevated levels of two IFN-inducible chemokine genes, including *CCL2* and *CXCL10*.^43^ *CCL2* (together with *CXCL10*) correlates with disease severity and mortality in patients with COVID-19.^44^, ^45^, ^46^, ^47^ Also, *CCL2* and *CCL3* are found to have significantly higher expression in patients with unfavorable outcomes compared to patients with successful recovery or healthy individuals.^46^ *CXCR1* and *CXCR2* chemokine receptors recruit mainly neutrophils. *CXCL2* and *CXCL5* are among the major neutrophil-attracting chemokines produced by lung epithelial cells during infection and they are highly expressed in patients with severe COVID-19 infection and associated with disease severity.^48^, ^49^

IL-5 is a type 2 cytokine that has rarely been studied in SARS-CoV-2 infection and may be anti-inflammatory in COVID-19 depending on the stage of the infection.^50^ Mehta et al. has shown that type 2 cytokines weaken early immune defense, and they do not contribute to hyperinflammation in COVID-19 patients' lungs.^51^ Moreover, type 2 cytokines may modulate the cytokine storm as these mediators can inhibit type 1 and type 17 immune response.^52^ According to research conducted by Agache et al., *IL-5* suppression exerts a positive effect on COVID-19 asthmatic patients.^53^ However, *IL-5* is not as effective as *IL-4* and *IL-13* in COVID-19.^50^

While our study identifies several differentially expressed lncRNAs and mRNAs associated with gender and comorbidities in COVID-19 patients, the underlying molecular mechanisms through which these transcripts contribute to disease severity remain largely unexplored. Further research involving functional validation studies, such as gene knockdown or overexpression experiments, as well as pathway-specific analyses, will be essential to elucidate how these regulatory RNAs influence immune responses, inflammation, and clinical outcomes. Additionally, investigating the interactions of these genes with sex-specific factors and specific comorbid conditions may uncover novel pathways that contribute to differential susceptibility and severity in COVID-19.

Investigating the role of lncRNAs in COVID-19, particularly in relation to gender and comorbidities, represents a relatively novel area of research. As the understanding of COVID-19 pathogenesis evolves, exploring how lncRNAs influence disease severity and interact with various biological and environmental factors is crucial. This study aims to contribute valuable insights into these dynamics, potentially revealing new therapeutic targets and enhancing our understanding of the complex interplay between genetic and non-genetic factors in COVID-19 outcomes. By addressing these gaps, our research may pave the way for more personalized approaches to treatment and prevention in affected populations.

## 5 Conclusion

The expression of mRNAs and lncRNAs in patients can provide insight into the effects of SARS-CoV-2 on specific parameters such as gender and comorbidities at the recovery and treatment stages. Different mRNAs and lncRNAs contribute to COVID-19 infection, but the mechanism is still under debate. In the present study, we analyzed DE-mRNA and DE-lncRNA interactions in four patient groups with different criteria, as provided in Table 1. Our results showed that the heme/hemoglobin metabolism pathway was common in three groups of patients (1, 2, and 4) and *ALAS2, AHSP, HBD, HBB, HBA1,* and *CA1* were common to all three datasets. In COVID-19 patients, an increase in the expression of these genes may act as a protective response to the hostile environment. Chemokines (*CCL2, CXCL2*, and *CXCL5*) are common to datasets 3 and 4. CCL2 may be involved in thrombo-inflammatory responses in severe COVID-19 and associated with adverse health outcomes and mortality in patients with COVID-19. *CXCL2* and CXCL5 may be helpful biomarkers for predicting subsequent neutrophil infiltration to determine an appropriate treatment as symptoms worsen. *IL-5* is a type 2 cytokine that may be anti-inflammatory in COVID-19 based on the stage of the infection.

In conclusion, the novelty of this study is analyzing mRNA and lncRNA expression in patients with different characteristics such as gender, underlying disease, and treatment or recovery stages. mRNAs and lncRNAs can be potential biomarkers to examine the severity of SARS-CoV-2 and determine treatment procedures. However, more studies are required to confirm the current research. It is, therefore, necessary to conduct a systematic review and a *meta*-analysis to establish robust evidence for these associations.

## CRediT authorship contribution statement

**Hassan Abolghasemi:** Supervision, Project administration, Conceptualization. **Hamidreza Kheiri:** Writing – original draft, Visualization, Investigation, Data curation. **Hamid Sedighian:** Investigation, Formal analysis. **Elham Behzadi:** Writing – review & editing, Writing – original draft, Investigation. **Reza Kachuei:** Writing – original draft, Investigation. **Mozhgan Kheirandish:** Writing – original draft, Investigation. **Masoud Arabfard:** Writing – review & editing, Visualization, Validation, Software, Methodology, Data curation. **Abbas Ali Imani Fooladi:** Writing – review & editing, Supervision, Project administration, Conceptualization.

## Informed consent

Not applicable.

## Ethics Approval and Consent to Participate

Not applicable.

## Funding

Not applicable.

## Declaration of competing interest

The authors declare that they have no known competing financial interests or personal relationships that could have appeared to influence the work reported in this paper.

## Data Availability

The datasets used and/or analyzed during this study are available from the corresponding authors upon reasonable request.
